# Using Biophysical Models to Understand the Effect of tDCS on Neurorehabilitation: Searching for Optimal Covariates to Enhance Poststroke Recovery

**DOI:** 10.3389/fneur.2017.00058

**Published:** 2017-02-23

**Authors:** Paola Malerba, Sofia Straudi, Felipe Fregni, Maxim Bazhenov, Nino Basaglia

**Affiliations:** ^1^Department of Medicine, University of California San Diego, La Jolla, CA, USA; ^2^Neuroscience and Rehabilitation Department, Ferrara University Hospital, Ferrara, Italy; ^3^Center of Neuromodulation, Spaulding Rehabilitation Hospital, Harvard Medical School, Boston, MA, USA

**Keywords:** stroke, tDCS, biophysical modeling, neurorehabilitation, motor recovery

## Abstract

Stroke is a leading cause of worldwide disability, and up to 75% of survivors suffer from some degree of arm paresis. Recently, rehabilitation of stroke patients has focused on recovering motor skills by taking advantage of use-dependent neuroplasticity, where high-repetition of goal-oriented movement is at times combined with non-invasive brain stimulation, such as transcranial direct current stimulation (tDCS). Merging the two approaches is thought to provide outlasting clinical gains, by enhancing synaptic plasticity and motor relearning in the motor cortex primary area. However, this general approach has shown mixed results across the stroke population. In particular, stroke location has been found to correlate with the likelihood of success, which suggests that different patients might require different protocols. Understanding how motor rehabilitation and stimulation interact with ongoing neural dynamics is crucial to optimize rehabilitation strategies, but it requires theoretical and computational models to consider the multiple levels at which this complex phenomenon operate. In this work, we argue that biophysical models of cortical dynamics are uniquely suited to address this problem. Specifically, biophysical models can predict treatment efficacy by introducing explicit variables and dynamics for damaged connections, changes in neural excitability, neurotransmitters, neuromodulators, plasticity mechanisms, and repetitive movement, which together can represent brain state, effect of incoming stimulus, and movement-induced activity. In this work, we hypothesize that effects of tDCS depend on ongoing neural activity and that tDCS effects on plasticity may be also related to enhancing inhibitory processes. We propose a model design for each step of this complex system, and highlight strengths and limitations of the different modeling choices within our approach. Our theoretical framework proposes a change in paradigm, where biophysical models can contribute to the future design of novel protocols, in which combined tDCS and motor rehabilitation strategies are tailored to the ongoing dynamics that they interact with, by considering the known biophysical factors recruited by such protocols and their interaction.

## Stroke Rehabilitation: Recruiting Changes in Brain Activity to Induce Mobility Recovery

### Cortical Activity after a Stroke

Stroke, due to the interruption of blood supply to the brain determining an acute neurologic condition (1), is a major cause of disability worldwide, often resulting in limited motor recovery in the paretic upper limb. Up to 75% of survivors maintain an arm paresis even in a chronic stage, with substantial limitations during participation in daily life activities (2, 3). The hemiparesis, in which the movement ability is affected on a single side, is due to the interruption of the motor signal through the corticospinal tract (CST) to the spinal cord motor neurons. Stroke research in humans is performed using techniques, such as functional magnetic resonance (fMRI) characterized by an excellent spatial resolution or transcranial magnetic stimulation (TMS), which explores cortical excitability through the induction of an electromagnetic field (4) at a high temporal resolution. Animal models of stroke have been influential in describing functional map reorganization (5) *via* electrophysiology, pharmacology (6), and optogenetics (7, 8).

A stroke initiates a large amount of changes in cortical excitability, connectivity (i.e., the synaptic wiring within and across brain regions), and ultimately coding (i.e., the specific neural spiking patterns that encode for movement are likely different after stroke). These changes, although not completely understood, occur on different time scales: some immediately after the injury and some are slowly established on the course of months (the chronic phase). However, times at which a stroke is considered entering the chronic phase, or exiting the subacute phase, are not universally agreed upon. Since measured changes in neural properties have been shown to affect the chances of motor recovery (9, 10), the design of effective neurorestorative approaches requires knowledge of the mechanisms of brain injury and neural repair after stroke.

Early after a stroke, cell deaths results from several biological pathways, including toxicity induced by excessive excitability, ionic imbalance, inflammation, and apoptosis. In an early response to stroke, several neurotrophic factors are upregulated, and in the first 1–4 weeks local axonal sprouting, dendritic spine expansion and synaptogenesis occur (11). In humans and animals, the affected brain areas (in particular the CST) show decreased activation in TMS studies (10), with concurrent activation of the contralateral cortex (12). Such reduced activity is related to increase in GABAergic tonic inhibition close to the lesion, which has been hypothesized to be neuroprotective in the acute phase, to counterbalance the excitotoxic cascade (13). At the same time, fMRI studies show that bilateral activation in both the ipsilesional (affected) and controlesional (unaffected) hemispheres occurs, revealing the development of early cortical reorganization processes (14, 15). These findings suggest that a damaged brain is still plastic and possibly amenable to be influenced by experiences.

In a chronic stage after stroke, a new functional cerebral architecture is determined, based on several variables (side of lesion, age, pre-stroke comorbidities). Since the disruption of the cortical motor network triggers a major reassembly of inter- and intra-areal cortical networks, it is reasonable that some of the functions of the injured regions could be redistributed across the remaining cortical and subcortical motor network in due time (12). In fact, several weeks after stroke, functional map changes are consolidated (16). Specifically, correlations between structural motor cortex connectivity and motor impairment (17) or fMRI activation in ipsilesional primary and pre-motor cortex and good upper limb recovery (15) have been highlighted, and impaired motor function seems related to persistent contralesional M1 activation (18). Though a rebalance between hemispheres is considered a sign of good recovery in chronic phase, whether such bilateral activation is adaptive or maladaptive is still on debate (19, 20).

### Recovery Depends on Network State

The progression of recovery can be seen as a relearning process of lost functions and as an adaptation and compensation of residual functions. Experimental animal data show that in absence of rehabilitation, functional spontaneous recovery occurs (21). However, it was limited and largely reflected the development of compensatory motor patterns far from normal kinematics.

The recovery process impinges on a damaged, reorganized network, and some of the changes in the acute to chronic phase after stroke can be predictive of rehabilitation outcomes. Even though a clear correlation between neurophysiological and neuroimaging findings and motor outcome in stroke survivors is not fully established, algorithms to predict motor recovery have been postulated (22). The imbalance between reduced excitability in the affected cortex and enhanced excitability of the unaffected hemisphere was predictive of motor recovery in a TMS study (9). In contrast, increases in contralesional primary motor cortex (M1) activity over the first 10 days after stroke correlated with the amount of spontaneous motor improvement in initially more impaired patients (23). Furthermore, repetitive training of the affected forelimb is related with a decreased motor representation of the intact hemisphere (24). These findings support the idea that after stroke an ipsilesional activity is rewired in patients with good recovery; whereas in patients severely impaired, the contralesional hemisphere can contribute to motor recovery.

Studies on animal models are essential to explore the time-windows more suitable to deliver rehabilitative interventions in order to achieve optimal neuroplastic changes (25, 26). For example, the upregulation of proteins occurs over a relatively narrow window of time after injury, which might be the optimal time to induce use-dependent cortical reorganization processes (16). Improvement in motor performance is associated with reorganization of cortical motor maps, but the temporal relationship between performance gains and map plasticity is not clear. Training-induced motor improvements are not reflected in motor maps until substantially later, suggesting that early motor training after stroke can help the evolving poststroke neural network (27). Also, in animals, a reduction of the increased tonic inhibition after injury induced functional recovery (6), which highlights the potential for animal models of stroke to provide new pharmacological targets.

### Use-Dependent Plasticity

Plasticity refers to an intrinsic property of the human brain to adapt to environmental pressures, physiologic changes, and experiences (28). Since experiences and practices play a fundamental role in neural reorganization processes both in healthy and damaged brains, neural plasticity is believed to be the basis for both learning in the intact brain and relearning in the damaged brain, which occurs through physical rehabilitation. Plasticity involves the brain at multiple system levels: intracellular (i.e., mitochondrial functions, genome changes), cellular (neurons and glia, including changes in synaptic strength, and sprouting), and network (changes in neuronal activation and cortical maps).

Motor behavior is extremely adaptive and may change during motor experiences. Skill training, which refers to the acquisition of new and complex movements’ combination, is able to induce cortical network reorganization, leading to increased synaptic number and strengths, and changes in the cortical topography closely related to the trained movement. These findings have been highlighted both in animals (29) and humans (30). It has been shown that cortical reorganization occurs if the tasks are challenging and quite new. In rat models, motor skill level increases rapidly over the first few days of skill training (31, 32), which are characterized by an increase in the synthesis of various proteins (including cAMP and the immediate early gene c-fos), and later phases of skill training are accompanied by significant increases in synapse number and motor map reorganization (31, 32). In humans, an intensive five-fingers motor training was able to modify significantly finger cortical motor maps (30).

Neuroscience research has made significant advances in understanding experience-dependent neural plasticity, and these findings are beginning to be integrated with research on the degenerative and regenerative effects of brain damage, leading to 10 experience-dependent plasticity principles by Kleim and Jones (33), which postulate that exercise to induce use-dependent plasticity should be intensive, task-specific, and salient from the patient perspective.

### Neurorehabilitation Techniques: Bottom-Up and Top-Down Approaches

**Figure 1:**
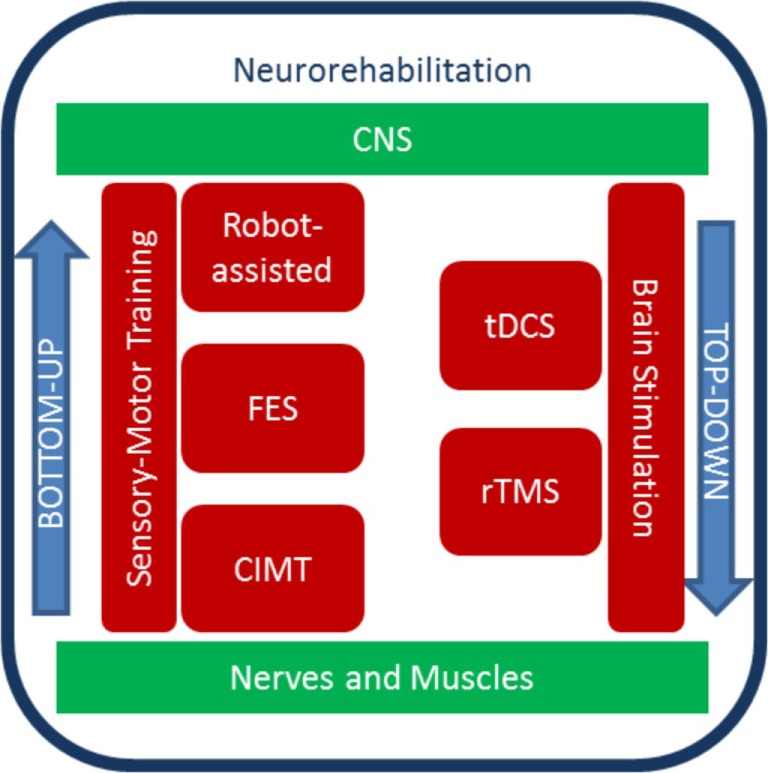
**Diagram of top-down and bottom-up stroke neurorehabilitation strategies**. Sensory–motor training and brain stimulation contribute to rehabilitation protocols that exploit neural plasticity. The bottom-up approach includes sensory–motor training, which can be aided by robots, electrical stimulation of the periphery, and constrains. The top-down approaches include methods to stimulate the brain non-invasively.

Even after damage to the central nervous system (CNS), a subject might achieve a functional recovery influenced by experiences and rehabilitative interventions. Recently, *bottom-up* and *top-down* approaches to enhance cortical reorganization and motor recovery after stroke have been introduced (34) (Figure 1). The former includes multimodal, external inputs that act at a peripheral level (bottom) with the aim of influencing CNS and neuroplastic changes. They are mainly represented by sensory–motor training. The latter enhance motor recovery with non-invasive brain stimulation (NIBS) techniques (35).

Within the bottom-up approaches, robotic devices have been developed for upper extremity stroke rehabilitation. To date, several studies (34, 36–39) highlight how robot-assisted therapy can improve arm motor function after stroke. Also, robots can be used to understand the stroke recovery process, such as the anticipatory control of arm movement (40) or motor synergies (41). Conversely, constraint-induced movement therapy (CIMT) uses limiting movements to promote recovery. Hemiplegic patients show inhibition of purposive movement of the affected arm in everyday life, mediated by compensation by the healthy arm (called “learned non-use”). CIMT promotes the reduction of this phenomenon, by inducing functional reorganization with repetitive, task-oriented practice (42) with the affected arm. Animal research shows that CIMT mediates unique functional reorganization processes of the CNS (43), and in stroke survivors, CIMT leads to improvement in real-world motor performances (44).

Electrical stimulation is used both in bottom-up and top-down approaches. Neuromuscular electrical stimulation generates joints movements by contracting muscles in an organized pattern, to generate functional movements such as hand grasping (45). This bottom-up modality is named functional electrical stimulation and has been shown to increase motor function (46) and cortical activation (47–49). In top-down strategies, NIBS techniques, such as repetitive transcranial magnetic stimulation (rTMS) or transcranial direct current stimulation (tDCS), are used to induce motor recovery in neurorehabilitation (50, 51). rTMS can modulate cortical plasticity and brain activity *via* the production of electromagnetic currents delivered by a coil placed over the scalp (52). tDCS applies weak direct currents to the scalp to modify cortical excitability for up to 90 min from the end of stimulation (53). It has advantages over rTMS, such as the greater portability and lower cost, the ability to stimulate both hemispheres simultaneously (54, 55), the long-lasting effects on cortical excitability with no significant adverse effects, and the lower level of discomfort experienced by patients. So far, tDCS effects on motor learning and arm function in stroke population have been extensively addressed testing the “interhemispheric competition model” where anodal tDCS is applied over the affected M1 and cathodal stimulation over the unaffected M1 (56). Moreover, tDCS can be combined with a behavioral training with an augmenting effect on motor learning (55, 57–59). Both rTMS and tDCS show large variability in the cortical excitability responses (60, 61), which emphasizes the need for a more deep understanding of how these neural stimulation techniques induce a response in the first place.

All these strategies are commonly applied in protocols which take advantage of the principles of use-dependent plasticity to induce functional recovery. However, definitive scientific evidence for the benefits of these restorative interventions is lacking so far. In addition, the neural mechanisms by which they may enhance recovery remain undefined.

### Clinical Hypothesis: Neural Dynamics during Neurorehabilitation Determines Motor Recovery

Overall, neurorehabilitation is a multifaceted process, which includes behavior and stimulation in an effort to conjure appropriate brain plasticity and mediate motor recovery. The aim of combining tDCS and motor training is to modulate the response of motor cortex area to a behavioral therapy *via* the modulatory effect of tDCS. So far, encouraging results have been shown in human studies (55, 57, 62). However, not all stroke patients improve equally after neurorehabilitation protocols.

Up to date, optimization of the effects of coupling tDCS and motor training still need further investigations. Presumably, important factors are the time since stroke, lesion site, the site and type of stimulation, the timing of stimulation in relation to physical intervention, and the motor task. tDCS stimulation can be delivered just before the motor task, priming functional networks for the physical intervention; during the behavioral intervention when it might preferentially interact with the networks selectively recruited by the ongoing task; or after motor training to promote a long-term consolidation of new neural pathways. Anodal tDCS delivered to M1 over the affected side was tested in subjects with chronic stroke in experimental designs (63, 64), and recently reviewed in Ref. (65, 66). Reduction of the excitability in M1 over the unaffected side with cathodal tDCS-promoted improvements in motor task lasted for the same amount of time (67). Recently, a bilateral tDCS montage has been proposed in stroke survivors to reduce interhemispheric inhibition (*via* cathodal stimulation over M1 unaffected) and to enhance cortical excitability (*via* anodal stimulation over M1 affected) with the final goal to decrease cortical excitability in the unaffected motor cortex and increase it in the affected motor cortex as demonstrated before (68).

**Figure 2:**
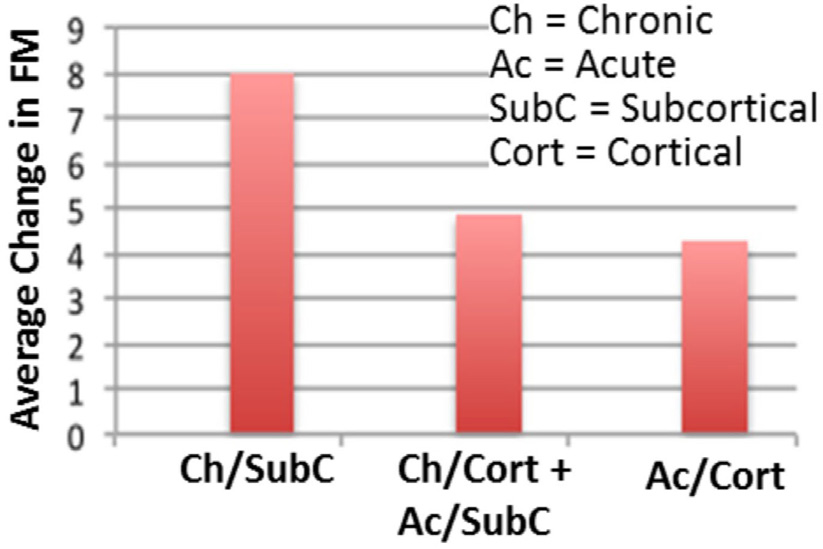
**Neurorehabilitation protocol with tDCS shows mixed results across stroke patient population**. For the same treatment, patients who had chronic subcortical stroke damage showed a higher recovery than those who had a different stroke type. Those who had the least improvement were patients with acute cortical stroke damage. Shown here is our data (58), which is one of many examples highlighting the need for optimized neurorehabilitation protocols, potentially different across stroke types and duration.

In our recent work (58), we employed such bilateral tDCS stimulation combined with upper extremity robot-assisted therapy on stroke survivors to reduce arm impairments, measured by a clinical assessment scale (69). Our findings suggested that bilateral tDCS was more effective for chronic stroke subjects with a subcortical lesion and less effective for patients in a subacute phase after stroke or with a cortical stroke (data shown in Figure 2). These findings are in line with previous studies that reported positive effects of bilateral tDCS on chronic stroke survivors when combined with CIMT (59) or with a meta-analysis that highlighted better tDCS results in chronic stroke population (50, 66, 70). Conversely, in an acute stage, all tried montages were found to be not effective in restoring motor function (71–73). Several reasons may explain differences among recovery stages: in the acute–subacute phase, the enhanced excitability of the intact hemisphere can be compensatory (74) and neuromodulation effects may be masked by spontaneous recovery. Also, in animal models, stroke training-induced motor improvements early after are not reflected in cortical reorganization until substantially later, suggesting that early motor training after stroke can help the evolving poststroke neural network (27). Regarding brain stroke localization, our findings are in line with a previous meta-analysis that found a larger effect size in subcortical stroke (75). Furthermore, variability in tDCS response has been shown to be strong among healthy individuals (60), and can contribute to the overall differences found in our data.

**Figure 3:**
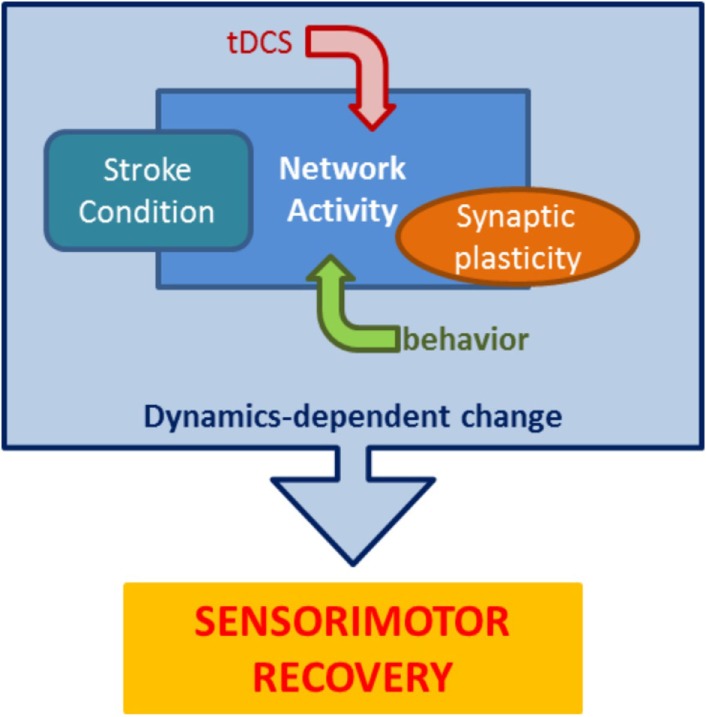
**The many components of stroke neurorehabilitation dynamics**. Stroke rehabilitation protocols impose goal-oriented behavior paired with tDCS on a poststroke network to recruit appropriate synaptic changes resulting in lasting improved performance. This schematic highlights the main components that interact during rehabilitation to mediate recovery.

Overall, these data suggest that incoming neurorehabilitation interacts with the specifics of network dynamics (excitability, plasticity, cortical map reorganization) that are depending on multiple factors, such as stroke location, time since stroke, and residual network architecture (76). This produces a picture like the one reported in Figure 3, where motor training, stimulation, synaptic plasticity, and the effects of stroke, all contribute to determining network dynamics during neurorehabilitation, ultimately shaping the potential for sensorimotor recovery.

## Modeling How Brain Dynamics Interacts with Stimulation and Neurorehabilitation

Understanding how motor rehabilitation and stimulation interact with ongoing brain dynamics is paramount to optimize rehabilitation strategies, but it requires theoretical and computational models to consider the multiple levels at which this complex phenomenon operates (77). Computational neuroscience is an emergent field of brain research which has been extremely successful in understanding both pathological [e.g., epilepsy (78, 79), Parkinsonism (80), Schizophrenia (81)] and normal [e.g., sleep (82–84), attention (85), sensory coding (86, 87)] brain processes. Computational modeling is most effective when the complexity of the problem and the underlying unknowns are vast, which is the case with stroke rehabilitation (88).

**Figure 4:**
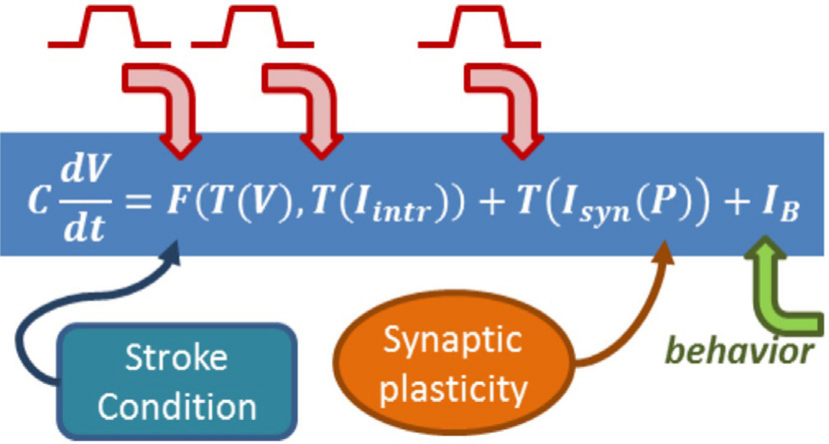
**Biophysical models include explicit equations for all the components influencing neurorehabilitation dynamics**. The equation summarizes a formalism in which the change of a cell voltage in time is equal to a non-linear function of the voltage itself and the state of intrinsic currents, summed to incoming synaptic currents. The formalism builds on Hodgkin–Huxley equations (89), which relate the dynamics of transmembrane channels in time (which will have their own equations) and how voltage changes in time. Intrinsic currents and synaptic currents (each with their own equations and variables) can be added once the formalism is in place. The arrows emphasize where in the biophysical model the role of behavior, synaptic plasticity, and stimulation can be included.

There are many different approaches when it comes to modeling brain activity, depending which level of detail is appropriate to represent a given phenomenon and introduce a possible explanation. In particular, biophysical models can include explicit variables and dynamics for damaged connections, changes in neural excitability, neurotransmitters, neuromodulators, and many different plasticity mechanisms; which together can represent a brain state, the effect of incoming stimulus, and task-related spiking activity (Figure 4).

In the following, we introduce the separate steps that compose the path toward a biophysical model of neurorehabilitation combined with NIBS, and for each step, we propose strategies to take advantage of what is known about stroke, tDCS, and neuroplasticity to design and adjust the parameters of a biophysical model.

### Modeling the Dynamics of M1 after Stroke

In a biophysical model of neural network activity, each cell is represented by its membrane voltage (changing in time) and a set of variables encoding the intrinsic cell dynamics (e.g., states of different intrinsic currents, like sodium, potassium, calcium, etc.). Cells are connected by synapses, with different expressions to represent excitatory and inhibitory connections, and synaptic currents are introduced by combining synaptic conductances with postsynaptic cell voltage. When the voltage of a presynaptic cell crosses a threshold, an action potential is generated and synaptic currents affect the voltage of all its postsynaptic cells (to hyperpolarize or depolarize for inhibitory or excitatory synapses, respectively). To represent synaptic plasticity, the strengths of synaptic conductances can be expressed as variables; with rules (equations) establishing changes in strengths, e.g., depending on timing of pre and postsynaptic cell spikes in the case of spike-timing dependent plasticity (STDP) (90). The overall network activity is then found by observing all cell spikes in time.

**Figure 5:**
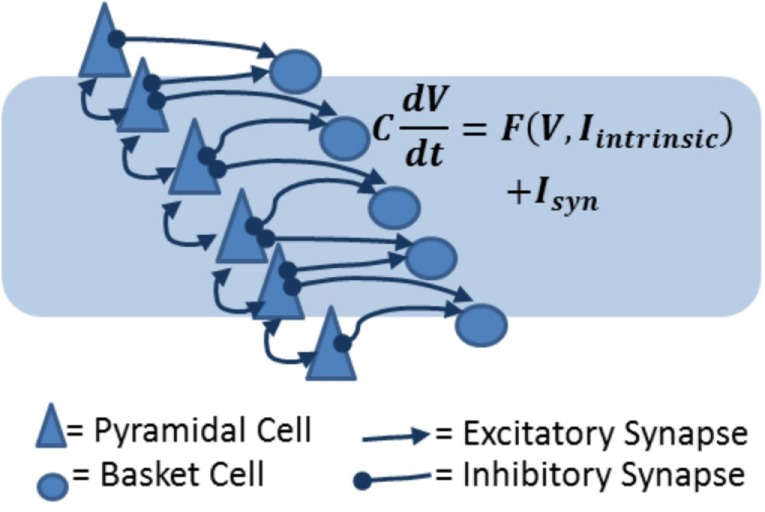
**Biophysical models of cortical layers include excitatory and inhibitory cells and synapses**. In the diagram, pyramidal cells are represented as triangles and basket cells are circles. Arrows represent excitatory connections (which can be AMPA or NMDA receptor mediated) and inhibitory (GABAergic) connections. The equation shows the basic principle on which the network model is constructed: each cell voltage changes in time according to the combined effect of intrinsic (voltage-dependent) currents and synaptic currents.

A model of stroke dynamics should minimally include excitatory pyramidal cells and inhibitory basket cells from different cortical layers. Within a layer, cells can be expressing AMPA, NMDA, or GABA_A_ synapses. In particular, a cortical layer should in general include feedback inhibitory (from basket cells to pyramidal cell) connections and recurrent excitatory (from pyramidal cells to other pyramidal and basket cells) synapses (Figure 5). Pyramidal cells in layer IV should show stronger recurrent connections. Across layers, pyramidal cells in layer II/III receive strong excitatory synapses from layer IV and deliver excitation to layer V pyramidal cells. Furthermore, pyramidal cells in layer IV will receive subcortical input, and the spiking activity of layer V pyramidal cells will be considered the output of the region, to be received and integrated by downstream structures (91–93), hence controlling, for example, motor response. This description is clearly a strong simplification compared to actual cortical anatomy, which includes a multitude of interneuron types (94) and subtle differences among pyramidal cells across and within layers (93). Nonetheless, the model design we suggest includes feedback inhibition and recurrent excitation within layers, and three separate processing layers across which subcortical input is converted into output.

**Figure 6:**
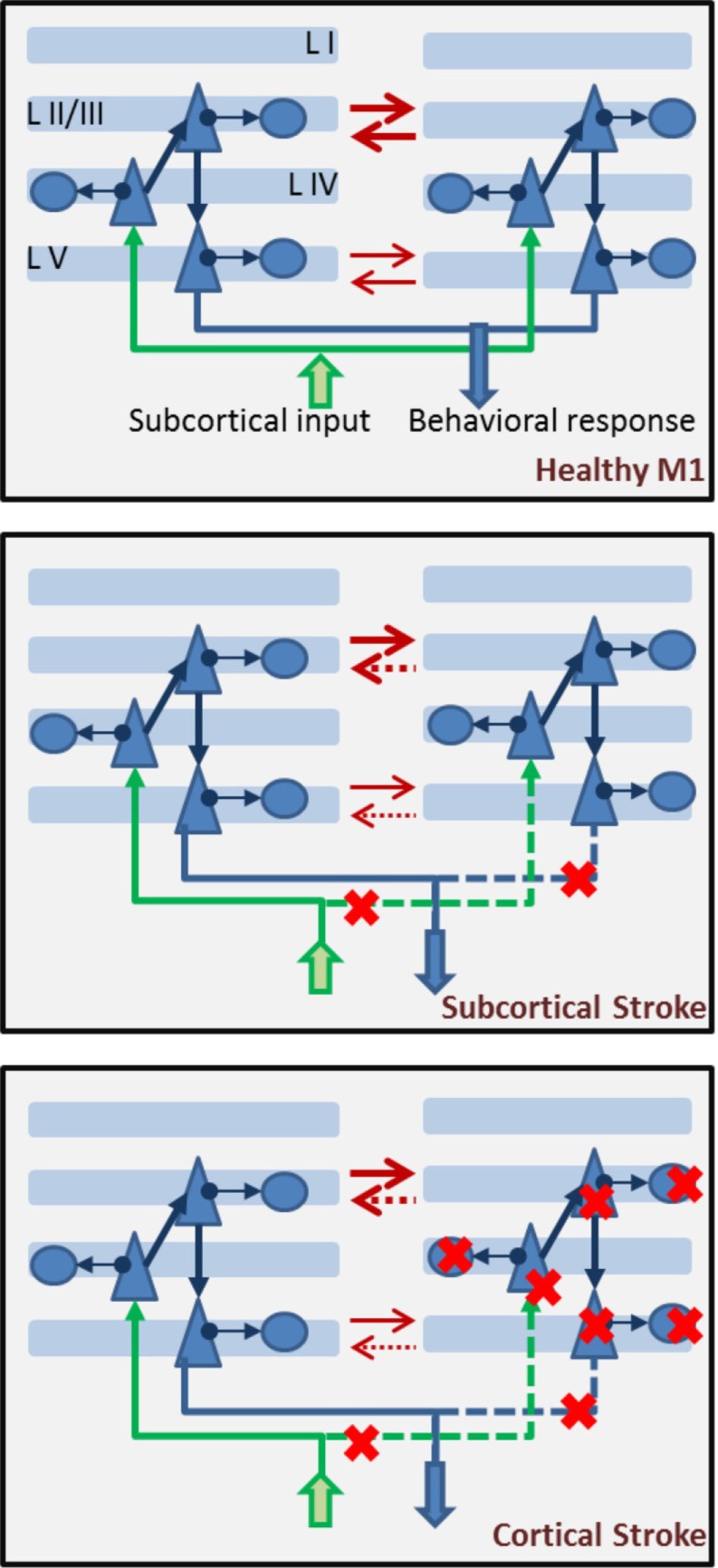
**Components of a biophysical model of stroke dynamics**. In the *top panel*, we show two hemispheres with multiple layers. Each layer, apart from layer I, is populated by pyramidal cells and basket cells, with connectivity as shown in Figure 5. Across layers, projections respect the canonical model of cortical connectivity: layer IV pyramidal cells receive input from subcortical regions (in green), they project to layer II/III pyramidal cells which in turn send their axons to layer V pyramidal cells where the cortical output is prepared and then projected toward other structures (in blue). The two hemispheres inhibit each other (red arrows) at both the layer II/III and layer V level (less strongly). In the *middle panel*, a subcortical stroke induces cell deaths among subcortical structures, leading to interrupted or strongly reduces input coming into the affected hemisphere (right side) and output being delivered to target regions. Red crosses mark the steps at which the flow of information is interrupted. Note that interhemispheric inhibition comes more strongly onto the affected hemisphere, which is suppressed by both augmented inhibition from a dis-inhibited unaffected hemisphere and reduced incoming subcortical input. In the *lower panel*, dynamics of the brain after a cortical stroke includes cell death (both excitatory and inhibitory) in the affected hemisphere. Red crosses mark the cells that could be removed. Severity of the damage will be represented by the percent of cells removed from the network. Note that interhemispheric inhibition is imbalanced in this case, as well as the subcortical case.

Typically, in a stroke event, one affected and one unaffected hemisphere are identified, and their competition/interaction is relevant to rehabilitation approaches (56, 95). Hence, a model of motor cortex which suffered a stroke event will include two hemispheres, connected by reciprocal inhibitory synapses, most dense between layers II/III and less dense between layers V of the two hemispheres. In a healthy version of the model, the two hemispheres will show identical properties. Conversely, the stroke will affect only one of the two hemispheres, and damage could be subcortical (leading to removal of incoming input to a portion of pyramidal cells in layer IV of the affected hemisphere) or cortical (leading to removal of a group of pyramidal and basket cells in the affected hemisphere, in addition to removed subcortical input) (Figure 6). The size of the damage, whether subcortical or cortical, can be represented by removing a different percent of the network incoming input and/or cell population in the hemisphere which suffered a stroke.

Consistently with the interhemispheric inhibition model of stroke (56), a network showing subcortical or cortical damage to one hemisphere should show dis-inhibited activity in the unaffected hemisphere, suppressing activity in the damaged one.

### Understanding the Effect of tDCS on Stroke Brain Dynamics

Another crucial step in this process is exposing the M1 model, in either the healthy or stroke-damaged condition, to non-invasive electrical stimulation, in our case tDCS. Most modeling efforts dedicated to tDCS have been concentrated on developing a detailed understanding of the current flow induced in the tissue by stimulation, using finite-element techniques [FEM (96–98)]. In fact, it is known that the reciprocal orientation of a pyramidal cell and the current field, it is immersed into factors into the possible polarization of the cell voltage (99–101). In a different approach, neural mass models have been introduced to address the comparison between the average activity of large groups of neurons and physiological (and clinical) measures of average changes induced by tDCS (53, 102). Finally, in few studies detailed biophysical models have been used to test effect of stimulation (103) and endogenous electric fields (104) on neuronal activity. However, even if research produced a perfect reconstruction of the current flow in the brain of a given patient exposed to a specific tDCS protocol, or an exact match between EEG measured in patients and predicted by a mass model, we would still be at a complete loss toward knowing what exactly was changed in network activity by incoming tDCS.

While the mechanisms that enable tDCS to induce lasting changes in network activity in humans and animals are not completely understood, research has uncovered a number of specific changes that tDCS imposes on brain tissue, and these can be applied to the design of a biophysical model. In fact, research has shown that tDCS is capable of inducing many changes: polarization of cell voltage (105, 106), scaling of synaptic gain (107) (where the efficacy of synapses can be locally enhanced or reduced), modulation of spontaneous firing (108), reduction of overall phasic GABA available to a cortical region (109), and control of the input–output function of pyramidal cells (110). All these are elements capable of modifying strongly not only the spiking of a single cell or a single synapse but the entire network activity profile. Considering the ability of tDCS to change these fundamental properties of neural activity, it is no surprise that tDCS can induce measurable changes in gamma synchrony (111), MEP amplitude (108), and ultimately performance in behavioral tasks (112).

In a biophysical model setting, membrane polarization, synaptic gain, cell input/output functions, and degree of intrinsic spiking of cells are all factors that can be explicitly changed by changing specific model properties (113, 114). Hence, each and every one of these effects of tDCS on the network can be studied separately or together. Furthermore, results about current flow in damaged tissue derived by FEM models (97, 98) can be used to make informed choices as to where in the network the effects of tDCS should be present, and how strong they should be.

### Representing Different tDCS Stimulations in a Model

Not all tDCS protocols are created equal, even if in the field of stroke rehabilitation a general consensus on the “interhemispheric inhibition” model has led to anodal stimulation delivered to the affected hemisphere, while cathodal stimulation is delivered to the healthy hemisphere, in an effort to promote re-balancing of the activation levels across the brain. Common variants across different tDCS protocols used in stroke motor rehabilitation are electrode placement and total charge, the latter then inducing scaling in waveform duration and intensity, hence ultimately affecting current density in the tissue, while the slope of current ramp-up (and down) can be used to obtain comparable data in sham/stimulation protocols. As mentioned before, the current field induced by tDCS has been thoroughly investigated with the use of FEM techniques, which have produced unexpected results which highlight the non-uniform effect that tDCS has on brain tissue (77). In a biophysical model, these different effects of stimulation can be taken into account by selecting which cells (and in which layers) will be changed by tDCS.

### Modeling How Synaptic Plasticity Mediates Long-lasting Changes in Brain Dynamics

Synaptic plasticity is a broad concept encompassing the many ways in which a synapse can change depending on network activity and signaling (115–117). The relevance of plasticity for learning (whether motor or otherwise) has been theorized in the Hebbian principle, where cells that spike in close time vicinity will show increased synaptic coupling, to promote a bond that would lead the two cells to preferentially firing together in the future (118).

Among the types of plasticity, STDP is the mechanism that most directly shows the Hebbian principle. In STDP, the time difference between a spike in the presynaptic cell and the postsynaptic cell controls the degree of change of synaptic strength: the smaller the time gap, the larger the change induced (90). If pre precedes post, the effect is to potentiate a synapse. Conversely, a synapse is depressed if post precedes pre. These changes can be short-lived or lasting for hours (or days). The latter case is called long-term potentiation or depression (LTP/LTD) (118). While the specifics of cellular mechanism that mediate LTP/LTD are not completely understood, it is common to associate LTP/LTD with strengthening/weakening of NMDA conductances (119).

Other plasticity mechanisms that could prove relevant in neurorehabilitation after stroke include heterosynaptic plasticity (120, 121), in which a synapse is strengthened/depressed depending on spillage of neurotransmitter from nearby synaptic clefts, and homeostatic plasticity (122, 123), in which the overall network activity influences the re-balancing of synaptic strength. Both heterosynaptic and homeostatic plasticity are needed to prevent “runaway synapses” (where synaptic weights distribution is strongly split between groups of very strong synapses reaching the ceiling of synaptic strength and very weak synaptic connections). Moreover, a number of neuromodulators (like ACh, dopamine, serotonine) can shape network plasticity, through reward/punishment signaling or promoting attention (115). Recent research in synaptic plasticity during and after tDCS has shown that long-term efficacy of tDCS requires NMDA signaling (124), ACh is necessary to increase spontaneous excitability with tDCS and modulate stimulation-induced LTP/LTD (125), and dopamine and serotonine have non-linear concentration-dependent influence on the lasting effects of tDCS (126–128). Furthermore, basic homeostatic plasticity seems to be involved in the effects of anodal tDCS, but not cathodal (129).

Biophysical models have standardized formalisms for the different synaptic plasticity mechanisms mentioned above. In basic STDP, the spike pattern of pre and post synaptic cell at a synapse and the strength of the synaptic conductance between the two cells are connected by an explicit relationship [see Ref. (82) for an example], and homeostatic plasticity can be expressed as synaptic scaling (79). The role of neuromodulators in plasticity can be introduced *via* reward/punishment components in STDP equations (130), or they can also play an indirect role, in affecting cell excitability rather than directly synaptic strength (131). Hence, a biophysical model of tDCS effect on stroke neurorehabilitation will likely need to include progressively more complex synaptic mechanisms, starting from STDP at NMDA synapses between pyramidal cells and pyramidal cells to interneurons, and progressing toward reward–punishment signals. Each plasticity mechanism (specific neuromodulators, heterosynaptic and homeostatic plasticity) can be introduced as needed, depending on the specific questions being addressed by the model.

Indeed, a similar approach was successfully applied to explore a novel hypothesis regarding role of homeostatic plasticity in epileptogenesis after brain trauma. It was shown using biophysical models that damage to the afferent inputs to the neocortex leads to disfacilitation and initial reduction of cortical activity. While homeostatic plasticity normally maintains a moderate level of activity in the cortex, it may fail to control excitability levels in heterogeneous networks, where there are subpopulations of neurons with severely different levels of activity—conditions found in traumatized cortex. In that case, upregulation of neuronal activity can lead to instabilities and epileptic seizures (132, 133).

### Representing Repetitive, Goal-Oriented Behavior in Network Dynamics

The overall factors that are influencing network dynamics during a stroke neurorehabilitation protocol revolve around one—or many—goal-oriented movements, repeated multiple times in each session. Hence, a repetitive task being performed during stimulation needs to be introduced in the biophysical model.

Repetitive behavior in the network (the task) can be represented by stimulating a group of cells multiple times, to induce a specific cell firing pattern (task pattern). For a task to be considered goal-oriented, it should recruit synaptic plasticity, potentially modulated by reward signals (130, 134). The test phase can be represented stimulating only a small fraction of the cells involved in the task and measuring the spike pattern evoked (test pattern). Performance can be then measured by the ability of the network to complete the pattern. This method has been successfully used in a number of models of behavioral experiments that included sequential activity (82, 130, 135). The difference between the test response and the task pattern can be quantified, to compare with the model that did not receive training.

More specifically, a subset of cells can be chosen to represent the activation sequence in M1 that would induce a “correct” movement response (task pattern), and those cells will receive short-lived subcortical inputs that cause them to spike in the correct order during training. Testing can rely on quantifying the degree of pattern completion when only a subset of cells in the task pattern is stimulated. In our specific case, the chosen measure can be scaled to match the range of the FM measure, which quantifies progress in the rehabilitation motor task considered.

### Combining All the Parts to Design a Model of tDCS and Neurorehabilitation Protocol for Stroke Recovery

**Figure 7:**
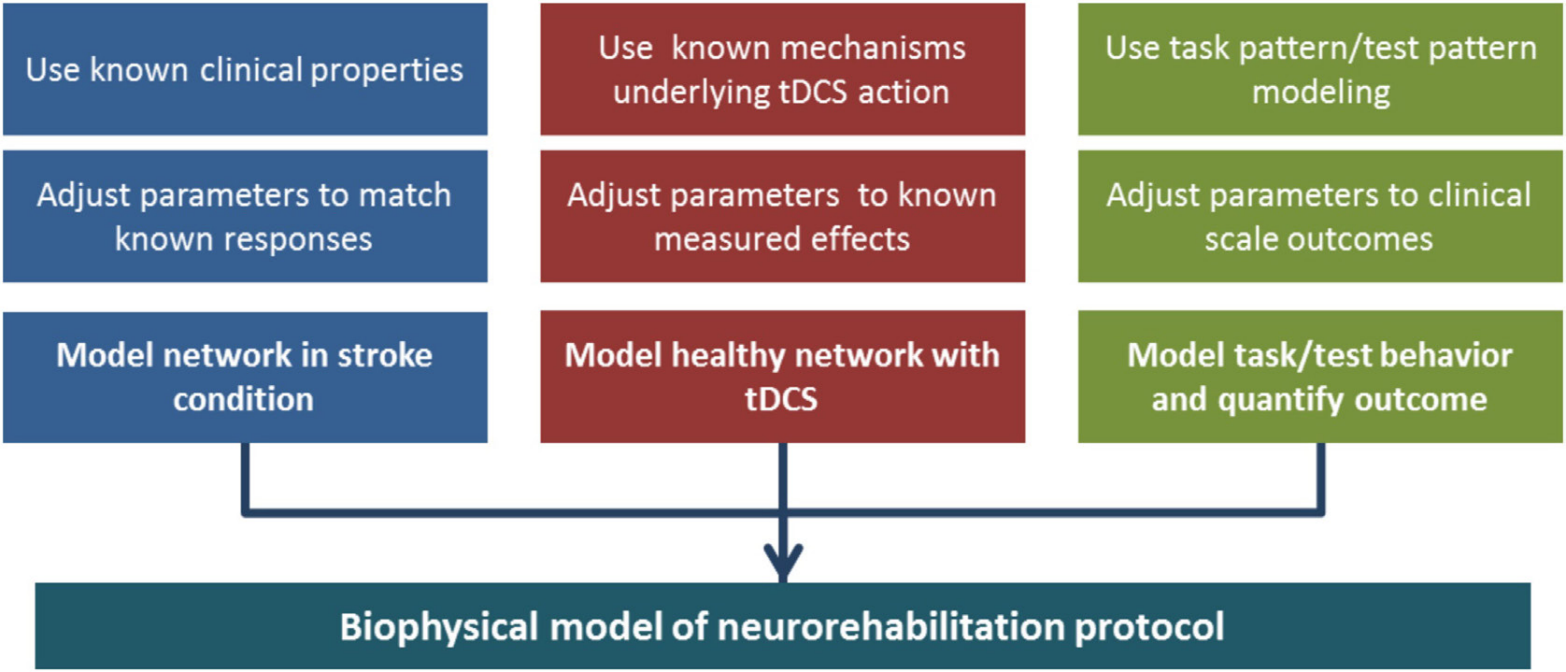
**Building a biophysical model of neurorehabilitation with transcranial direct current stimulation (tDCS)**. In the diagram, we emphasize the three main components that are necessary to build the full model. The different branches can take advantage of knowledge accumulated in the separate fields of research in tDCS effects, stroke anatomy, and pattern formation/completion. Each component can be tuned to experimental data, and three separate models can be merged in a complete, albeit complex, unified model.

While above we have listed the many pieces that compose this puzzle, they are only means to the goal of assembling a unified computational model, which could be progressively built on the smaller components that we identified here. The process can be articulated in parallel developments (Figure 7): a model of two hemispheres, with cortical layers and the ability to represent healthy and stroke-affected activity, a model of tDCS affecting the biophysics of a network, changing excitability, synaptic strengths, and recruiting plasticity, and a model of goal-oriented learning, where task and test patterns are defined and measures of performance in the model are related to clinical outcomes. The three lines then can merge in one objective: a model of the dynamics of stroke neurorehabilitation with stimulation.

## Biophysical Hypothesis: tDCS Can Promote the Role of Phasic Inhibitory Signal on Plasticity

Neurorehabilitation is based on facilitating relearning of motor skills, which is mediated by the many forms of synaptic plasticity. An effective rehabilitation protocol will induce spiking activity able to code for movement, and promote its consolidation. A balanced excitatory/inhibitory background activity, mediated by a combination of feed-forward excitation and feedback phasic inhibition (136), guarantees that cortex can process stimuli without runaway excitability (137). In other words, a balanced inhibitory tone allows cortex to compute effectively, while too low or too high inhibitory signal interferes with network coding. After a stroke, the level of inhibitory tone is de-regulated, and effective recovery promotes returning to a functional equilibrium. In general, lost connections after stroke would prevent a return to exactly the same conditions, but increased efficacy of residual connections can compensate for lacking ones, at least within a range.

While, in all strokes, a degree of imbalance in the interhemispheric inhibition is present and increased tonic GABA signaling is expected, the changes in phasic GABA signals seem to be more subtle. Recent work (138) on animal models shows that GABA receptors present at perisomatic synapses on pyramidal cells increase their efficiency during stroke recovery. Interestingly, this enhancement was found to be constrained to layer V, which is where information is finally processed before being translated into behavioral response. Increased phasic GABA at layer V pyramidal cells synapses is considered to contribute positively to recovery, because when phasic GABA efficacy was increased pharmacologically with Zolpidem, recovery was accelerated. It is, therefore, plausible that when tDCS facilitates neurorehabilitative interventions, it recruits activity to effectively increase phasic GABA signaling on layer V pyramidal cells. In this framework, the mechanism by which tDCS could promote recovery of network function could be different, depending on the circuitry it impinges on, hence depending on the specifics of the stroke event.

If one of the mechanisms involved is the phasic inhibition on layer V pyramidal cells, it can be recruited by driving local inhibitory neurons to spike, through excitatory synaptic inputs. Excitatory cells in layer V are able to trigger local feedback inhibition, and translaminar connectivity can also drive feed-forward inhibition, in particular through projections from pyramidal cells in layer IV to layer V fast spiking interneurons (139). Note that input from layer II/III neurons seems to preferentially target layer V interneurons which route inhibition to the apical dendrites of the target cells, and so it would not be capable to recruit phasic somatic inhibition in layer V.

In subcortical strokes, layer IV pyramidal cells have their input from subcortical systems reduced, the loss of incoming excitatory input leads to disfacilitation and activity in the layer decreases [in a process similar to immediate the reduction of slow oscillation activity in deafferented cortex (140)]. In time, homeostatic compensation mechanisms can induce an enhanced excitability in pyramidal cells, which results in an imbalanced network state. When the missing afferent signals are compensated by tDCS (albeit from superficial cortex stimulation), the cortical network recovers an excitability state which is balanced, which contributes to balancing the local phasic inhibitory tone. Hence, use-dependent plasticity promoted during tDCS stimulation tends to consolidate a network state able to relearn, including potentially enhancing phasic inhibitory activity in layer V.

Conversely, in cortical strokes it is possible that the local circuitry is too damaged to find a newly effective coding state in the presence of a general shift toward excitability as induced by locally applied tDCS. This could explain why using identical protocols in neurorehabilitation with tDCS has different effects on subcortical and cortical stroke: while in the subcortical case generalized drive can help the local network recover a balanced state by promoting a recovery of the phasic inhibitory feedback, in cortical strokes the local network is too damaged, and the same tDCS intervention cannot induce enough re-balancing of excitatory/inhibitory signal.

## Changing the Paradigm: Using Brain Dynamics to Optimize Protocols

It is important for the stroke rehabilitation field to embark in research that relies upon appropriate biophysical modeling of network dynamics. While FEM models are bridging between brain connectomics and tDCS input, and mass models are connecting clinical data to the average spiking in brain regions, biophysical models have the ability to build on these findings, contributing to a higher detail, and offering tools to investigate separately all the effects of behavior, stimulation, and stroke condition. In fact, simple biophysical models of tDCS applied to decision-making are already producing result (135).

In this work, we have shown the principles that can define an overall biophysical model capable to undergo a rehabilitation protocol and highlighted the different complexity levels that can be introduced depending on the specific hypothesis under investigation, for example introducing reward among plasticity mechanisms to represent goal-oriented physical training. A lot of knowledge in stroke neurorehabilitation and tDCS effects can pour in a properly designed biophysical model, while parameters can be adjusted by comparing to known clinical outcomes, and measured effects on the network. Subsequently, different models—modified appropriately—can make predictions about the efficacy of a given rehabilitation protocol, and which mechanism(s) might underlie the overall performance improvement—or lack thereof.

The mixed results brought by current tDCS neurorehabilitation protocols shed a light on the open questions underlying motor recovery after stroke. At present, however, we can only suggest that available data points toward the necessity of different protocols for different stroke types. As biophysical models are developed, these questions can find new ways of being addressed, including possibly the design of novel tDCS protocols to pair with motor neurorehabilitation, based on model predictions. This is a particularly appropriate time to start introducing computational modeling of brain dynamics into stroke rehabilitation research, because progress is ongoing into the specific effects that tDCS has on a cortical network, and data connecting stroke state to quantifiable measures, such as TMS and high-density EEG, is available in large numbers. These progresses on the clinical and basic science side will now make possible the design and implementation of a new computational neuroscience of stroke neurorehabilitation.

## Author Contributions

PM and SS designed research and wrote the paper, FF, NB, and MB critically revised the manuscript, suggested improvements, and consulted on research design.

## Conflict of Interest Statement

The authors declare that the research was conducted in the absence of any commercial or financial relationships that could be construed as a potential conflict of interest.
